# Readthrough Acetylcholinesterase Is Increased in Human Liver Cirrhosis

**DOI:** 10.1371/journal.pone.0044598

**Published:** 2012-09-13

**Authors:** María-Salud García-Ayllón, Cristina Millán, Carol Serra-Basante, Ramón Bataller, Javier Sáez-Valero

**Affiliations:** 1 Instituto de Neurociencias de Alicante, Universidad Miguel Hernández-CSIC, San Juan de Alicante, Spain; 2 Centro de Investigación Biomédica en Red sobre Enfermedades Neurodegenerativas (CIBERNED), San Juan de Alicante, Spain; 3 Unidad de Investigación, Hospital General Universitario de Elche, Elche, Spain; 4 Liver Unit, Hospital Clínic, Institut d'Investigacions Biomèdiques August Pi i Sunyer (IDIBAPS), Barcelona, Spain; 5 Centro de Investigación Biomèdica en Red de Enfermedades Hepáticas y Digestivas (CIBERehd), Barcelona, Spain; Weizmann Institute of Science, Israel

## Abstract

**Background & Aims:**

There have been many studies on plasma butyrylcholinesterase in liver dysfunction. However, no data is available about acetylcholinesterase in human cirrhosis, although profound changes have been described in cirrhotic rat models.

**Methods:**

Human serum and liver acetylcholinesterase and its molecular forms were determined enzymatically, after butyrylcholinesterase immunodepletion. The distinct species of acetylcholinesterase, with a distinct C-terminus, were determined by western blotting, and the level of liver transcripts by real-time PCR. Liver acetylcholinesterase was also evaluated by immunocytochemistry.

**Results:**

In patients with liver cirrhosis, the activity of plasma acetylcholinesterase (rich in light species), appeared to be apparently unaffected. However, the levels of the soluble readthrough (R) acetylcholinesterase form, an acetylcholinesterase species usually associated with stress and pathology, was increased compared to controls. Human liver acetylcholinesterase activity levels were also unchanged, but protein levels of the acetylcholinesterase-R and other acetylcholinesterase subunit species were increased in the cirrhotic liver. This increase in acetylcholinesterase protein expression in the cirrhotic liver was confirmed by PCR analysis. Immunohistological examination confirmed that acetylcholinesterase immunoreactivity is increased in parenchymal cells of the cirrhotic liver.

**Conclusions:**

We demonstrate significant changes in acetylcholinesterase at the protein and mRNA levels in liver cirrhosis, with no difference in enzymatic activity. The altered expression of acetylcholinesterase protein may reflect changes in its pathophysiological role.

## Introduction

Cholinesterases are a family of ubiquitous enzymes frequently studied in association with many pathological processes. Acetylcholinesterase (EC 3.1.1.7; AChE), the enzyme chiefly responsible for the inactivation of cholinergic neurotransmission, has been associated to cognitive dysfunction in Alzheimer's disease [1], and to disorders such as neuromuscular dysfunction and myasthenic syndromes, tumorigenesis among many others (for a review see [2], [3], [4]). AChE is present in serum and liver, however its physiological significance other than inactivating acetylcholine has thus far not been elucidated. Thus, roles for AChE other than its acetylcholine hydrolytic activity have been proposed [2], [3], [4]. A second cholinesterase, butyrylcholinesterase (EC 3.1.1.8; BChE), whose physiological role is also unknown, co-exists with AChE in many tissues, including those with no cholinergic function [2], [5]. The two cholinesterases are regulated by separate mechanisms [6], [7]. Although very few studies have addressed the levels of cholinesterase in human liver [8], [9], it is widely accepted that plasma BChE originates in liver cells and represents the major cholinesterase in human serum, approximately 160 times higher than AChE [10]. Consequently, previous studies on cholinesterase changes during liver dysfunction have focussed on serum BChE; whereas AChE has not received much attention. Indeed, the use of serum BChE activity as an indicator of liver function has been employed for decades [11].

So far, the study of AChE in pathological liver has been restricted to hepatocellular carcinoma [12] or to animal models that have less BChE than AChE activity [13]. Using the rat as an animal model with low serum BChE activity, we have reported that AChE is significantly altered during liver cirrhosis [13]. However, the contribution of AChE and BChE and their different molecular forms varies between rats and humans, and to date, there is no data on potential alterations of AChE expression in the cirrhotic human liver.

Classical studies on cholinesterase usually focus on enzymatic activity and molecular forms of the enzyme. AChE occurs as both active and inactive subunits [14], [15], [16]. Different AChE species are derived from alternative RNA splicing, generating different polypeptide transcripts with the same catalytic domain, but with distinct C-termini. It would be useful to perform further western blot analyses using different anti-AChE antibodies raised against different C-terminal peptides. In this study, we have measured AChE activity in non-diseased and cirrhotic human liver and plasma after BChE removal by immunoprecipitation. We have compared the different molecular forms and subunit banding pattern of AChE by SDS-PAGE under reducing conditions followed by Western blotting. AChE expression was assessed by quantitative RT-PCR analysis of the different AChE mRNAs, and the distribution of AChE protein was investigated in normal and cirrhotic human liver by immunohistochemistry.

## Patients and Methods

### Patients

For this study we obtained ethics approval from the ethics committee at our institutions (Universidad Miguel Hernández, Elche, and Hospital Clínic, Barcelona) and obtained written informed consent from all involved participants. The study was carried out in accordance with the Declaration of Helsinki.

Plasma samples from patients with liver cirrhosis and age-matched controls were provided by the Hospital General Universitario de Alicante (Spain), as previously described [17]. Causes of cirrhosis were alcoholism, Hepatitis C virus (HCV) infection and both alcoholism and HCV. Plasma was separated from whole blood by centrifugation, aliquoted and frozen at −80°C until use.

Liver samples were obtained from the Hospital Clinic of Barcelona (Spain) and collected as described in a previous study [18]. Fragments of normal liver adjacent to colon cancer metastasis and liver specimens obtained immediately after laparotomy and before vascular clamping were collected as control cases. All controls had normal serum aminotransferase levels and normal liver histology. Cirrhotic human samples were obtained from liver explants after transplantation in patients with chronic HCV-induced liver disease, from percutaneous liver biopsy of patients with detectable serum RNA HCV and increased alanine aminotransferase levels, and from resections of hepatocarcinoma in cirrhotic patients.

### Tissue homogenization and cholinesterase extraction

For cholinesterase extraction, small pieces of liver stored at −80°C were thawed slowly at 4°C and homogenized (10% w/v) in ice-cold Tris-saline buffer (50 mM Tris-HCl, 1 M NaCl, and 50 mM MgCl_2_, pH 7.4) containing 0.5% (w/v) Triton X-100 and supplemented with a cocktail of proteinase inhibitors [19]. The suspension was then centrifuged at 100,000×g for 1 hr at 4°C to recover a cholinesterase rich fraction.

### BChE immunoprecipitation

In plasma and liver samples BChE was immunoprecipitated by two successive incubations using an anti-BChE coupled affinity resin (protein A-Sepharose with an anti-BChE polyclonal antibody generously provided by Prof. Oksana Lockridge, University of Nebraska Medical Center, Omaha, NE, USA), as previously described [10]. The two successive incubations with the anti-BChE resin ensured that the majority of the BChE activity in the samples was removed. Plasma and liver samples immunodepleted of BChE were then used for AChE determination.

### Enzyme assays and protein determination

AChE and BChE activity were determined by a modified microassay method of Ellman [10]. AChE was assayed with 1 mM acetylthiocholine and 50 µM tetraisopropyl pyrophosphoramide (Iso OMPA), a specific inhibitor of BChE; while BChE was measured with 1 mM butyrylthiocholine and 10 µM BW284c51, a specific inhibitor of AChE. One milliunit (mU) of AChE or BChE activity was defined as the number of nmoles of acetylthiocholine or butyrylthiocholine hydrolysed per min at 22°C. Protein concentrations were determined using the bicinchoninic acid method, with bovine serum albumin as standard (Pierce, Rockford, IL).

### Sedimentation analysis

Molecular forms of AChE and BChE were separated according to their sedimentation coefficients by centrifugation on 5–20% (w/v) sucrose gradients containing 0.5% (w/v) Triton X-100, as previously described [20]. Approximately 40 fractions were collected from the bottom of each tube and assayed for cholinesterase activities. The cholinesterases present in liver and plasma are mainly tetramers (G_4_) and light globular species (dimers, G_2_, and monomers, G_1_).

### Detection of AChE by Western Blotting

AChE subunits were detected by immunoblotting under denaturing and reducing conditions. As plasma samples contain high amounts of certain plasma proteins (albumin, immunoglobulins, transferrin etc.), these were first depleted by immunoaffinity-based chromatography (Seppro® IgY14 spin column kit, GenWay Biotech Inc, San Diego, CA) prior to electrophoresis. Samples from plasma (25 µg of protein after protein depletion) and liver (50 µg) were resolved by electrophoresis on 10% SDS-polyacrylamide slab gels. Following electrophoresis, proteins were blotted onto nitrocellulose membranes, blocked with 5% bovine serum albumin and incubated overnight with different anti-AChE antibodies: N-19 (Santa Cruz Biotechnology), Ab31276 (Abcam, Cambridge, UK), an antibody raised to the unique C-terminus of human AChE-R ([21], a generous gift from Prof. Hermona Soreq, The Institute of Life Sciences, The Hebrew university of Jerusalem, Jerusalem, Israel), and T548, a polyclonal rabbit anti-mouse AChE antibody raised against a recombinant AChE protein made in mammalian HEK cells and corresponding to mouse AChE sequence truncated at residue 548 ([22], generously provided by Prof. Palmer Taylor; Department of Pharmacology, Skaggs School of Pharmacy and Pharmaceutical Sciences, University of California, San Diego, La Jolla, California, USA). Nitrocellulose strips were then incubated with HRP-conjugated secondary antibodies (Santa Cruz Biotechnology) and immunoreactive AChE was detected using the ECL-Plus kit (Amersham Life Science, Arlington Heights, IL) in a Luminescent Image Analyzer LAS-1000 Plus (FUJIFILM). Molecular weight markers were used to determine protein size (Sigma-Aldrich Co, St. Louis, MO). For semi-quantitative analysis, the intensity of AChE bands was determined with the Science Lab Image Gauge v4.0 software provided by FUJIFILM.

### RNA isolation and analysis of AChE transcripts by QRT-PCR

Total RNA was extracted using the TRIzol reagent (InvitrogenTM, Carlsbad, CA) according to the manufacturer's protocol. Five hundred nanograms of RNA were retro-transcribed using a high capacity complementary DNA reverse transcription kit (Applied Biosystems). Quantitative PCR amplification was performed using a StepOne™ Real-Time PCR System (Applied Biosystems, Foster City, CA) with Power SYBR® Green PCR Master Mix according to the manufacturer's instructions (see primer sequence in the legend of Fig. 3). Transcript levels for AChE (R, T and H transcripts) were calculated using the relative standard curve method normalized to GAPDH.

### AChE immunocytochemistry

Paraffin-embedded 3 µM liver sections were incubated with goat anti-human AChE (N19, 1∶100, Santa Cruz Biotetechnology, SC6431) overnight. After extensive washing with PBS, sections were incubated with a polyclonal rabbit anti-goat antibody conjugated to HRP (1∶600, Dako, E0466). Following further washing in PBS, a secondary goat anti-rabbit antibody conjugated to HRP (DAKO EnVision System-HRP, K4007) was added for 30 minutes at room temperature. 3,3′ diaminobenzidine (DAB, Dako) was used as a chromogen, and sections were counterstained with hematoxilin. As negative controls, all specimens were incubated with isotype-matched primary antibodies.

### Statistical analysis

Measurements are expressed as means ± SEM. Data was analyzed by a Student's t-test (two tailed) for single pair-wise comparisons using SigmaStat (Version 2.03; SPSS Inc.) software. Statistical significance was designated as *p*<0.05.

## Results

**Figure 1:**
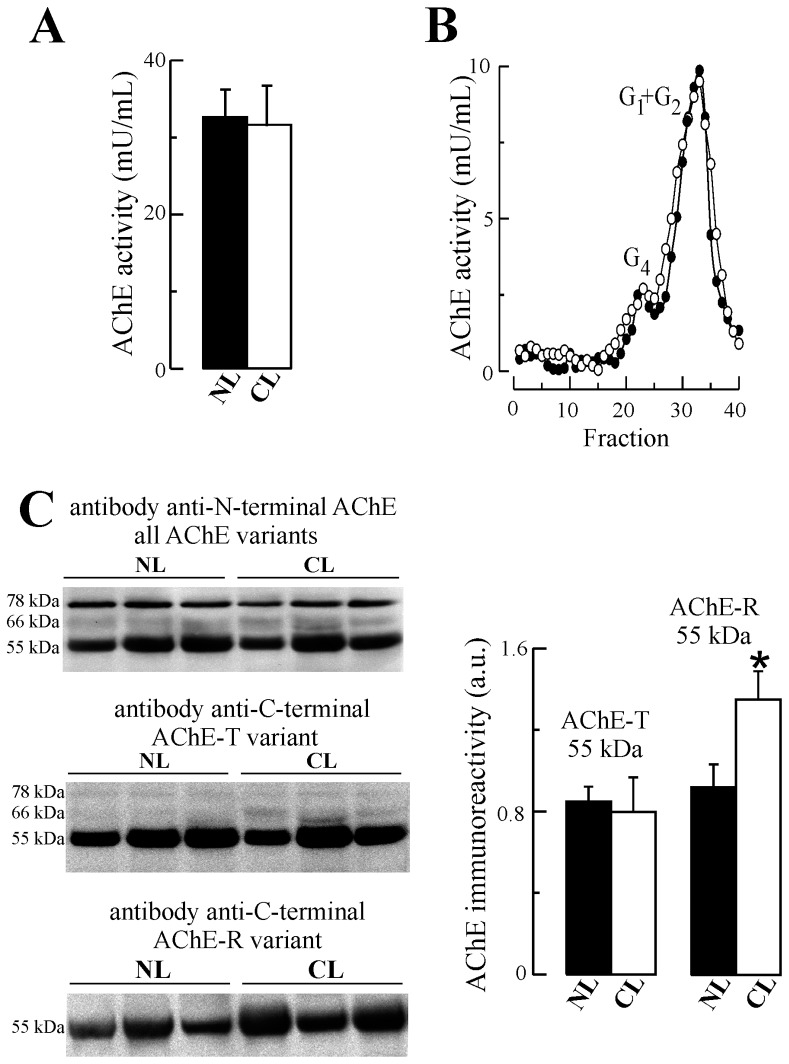
Increased levels of the acetylcholinesterase-R (AChE-R) in plasma from individuals with cirrhotic livers. Plasma from non-diseased controls (NL; n = 12, •) and cirrhotic individuals (CL; ○; n = 8) was immunoprecipitated with anti-BChE antibody and total AChE activity levels (**A**) and AChE molecular forms levels (monomers and dimmers: G_1_+G_2_) (**B**) were determined after immunoprecipitation. (**C**) Immunodetection of AChE using denaturing conditions was performed with an antibody raised against the N-terminus of AChE, common to all AChE subunits. For densitometric quantification, antibodies raised to the unique C-terminus of AChE-T and AChE-R subunits were used. a.u.: arbitrary units. Columns represent means ± SEM. *p<0.05.

AChE and BChE activities are routinely measured in the same extract using specific substrates and inhibitors. However, the elevated levels of BChE in human plasma are expected to interfere in the determination of AChE, even in presence of BChE inhibitors [13]. Thus, in order to estimate subtle changes in plasma AChE levels from patients with cirrhotic liver conditions, we measured AChE activity in plasma, after two cycles of BChE immunoprecipitation [13]. No apparent differences in AChE activity were observed in plasma from cirrhotic patients compared to controls (Fig. 1A). BChE activity levels were lower in CL samples prior to immunodepletion compared to controls (34% decrease; *p* = 0.008) (Fig. S1A).

Both AChE and BChE are expressed as several molecular forms distinguishable by their molecular weights [23]. Plasma supernatants before and after BChE-immunodepletion were fractionated on sucrose density gradients to separate the different AChE molecular forms. No difference was observed in the amount of the major G_1_+G_2_ AChE species, nor in the minor G_4_ form from CL plasma compared to controls (Fig. 1B). In undepleted plasma, the major G_4_ form of BChE displayed a decrease in levels in parallel with a decrease in total BChE activity (Fig. S1B).

Isoforms of AChE containing the same catalytic domain, but with distinct C-terminal peptides, are generated by alternative RNA splicing [4], [23]. This alternative splicing is differentially regulated in different cell types, at both the mRNA and post-translational level. We have recently demonstrated the presence of type T (‘tailed’) and type R (‘readthrough’) AChE subunits in human plasma using different anti-AChE antibodies raised against different C-terminal peptides. The third subunit, type H (‘hydrophobic’) is not able to be detected due to the lack of specific antibodies [10]. We have also analyzed the complex banding-pattern of the different AChE subunits by SDS-PAGE/Western blotting using these different anti-AChE antibodies (Fig. 1C). The antibody N-19, raised against a peptide that maps to the N-terminus of human AChE, common to all subunits, detected three major bands of approximately 78, 66 and 55 kDa. An alternative anti-AChE antibody, T548, which specificity has recently demonstrated in brain extracts from wild-type and knockout AChE mice [24], allowed us to ensure the true identity of lighter AChE subunits (Fig. S2). A similar pattern of AChE labelling was demonstrated with an alternate anti-AChE antibody ab31276, which recognizes the C-terminal of the AChE T subunits. No difference in immunoreactivity of the predominant and more clearly defined 55 kDa subunit was observed between pathological and non-diseased samples (Fig. 1C). Interestingly, the antibody to the R-AChE subunits, directed to the unique C-terminus of AChE-R, and which only detects a 55 kDa band similar in size to the T-subunit, displayed a significant increase in immunoreactivity in cirrhotic samples (47% increase; *p* = 0.03) (Fig. 1C).

**Figure 2:**
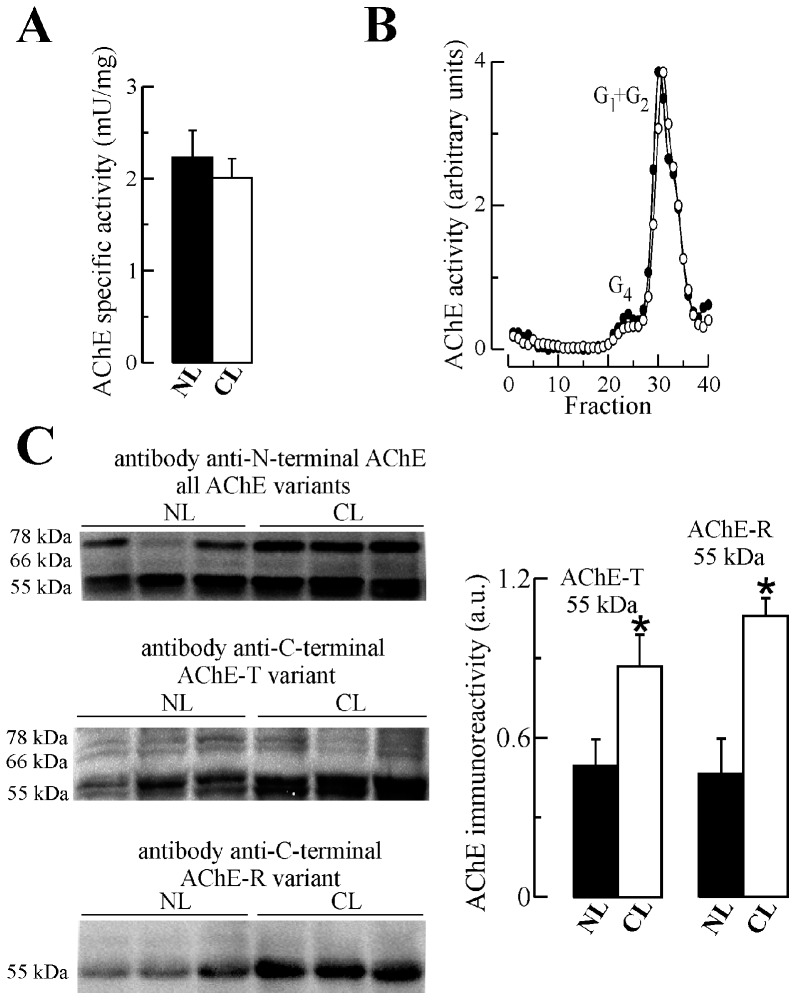
Increased levels of acetylcholinesterase (AChE) protein levels in liver extracts form cirrhotic individuals. Liver extracts from non-diseased (NL; ▪; n = 6) and cirrhotic liver cases (CL; □; n = 6) were immunodepleted of major butyrylcholinesterase (BChE) activity, and total AChE activity levels (**A**) and AChE molecular species (monomers and dimmers: G_1_+G_2_) (**B**) were determined after BChE depletion. (**C**) Samples were immunoblotted under denaturing conditions with different anti-AChE antibodies. The N-terminal antibody, which recognizes all AChE subunits, and antibodies directed to the different C-termini of the AChE species (AChE-R and T subunits), were used for densitometric quantification. * *p*<0.05.

The levels of BChE and AChE after BChE immunodepletion were measured in the liver of cirrhotic patients to examine changes in cholinesterase levels. Despite a decrease in BChE in the cirrhotic liver (50% decrease; *p* = 0.002; Fig. S3A), no significant change in AChE levels was observed (Fig. 2A). Tetrameric and light molecular species of BChE were both decreased in cirrhotic individuals (Fig. S3B); whereas the amount of monomeric AChE was indistinguishable from amounts in the control group (Fig. 2B). When liver extracts of AChE were analyzed by Western blotting using the N-19 antibody, the three typical AChE bands (55, 66 and 78 kDa) were characterized (Fig. 2C). Immunoblotting with the ab31276 antibody, specific for AChE T subunits, demonstrated an increase in the 55 kDa immunoreactive band in cirrhotic extracts (119% increase; *p* = 0.03; Fig. 2C). In cirrhotic samples, the antibody to R-AChE also displayed a significant increase in immunoreactivity (128% increase; *p*<0.001) (Fig. 2C).

**Figure 3:**
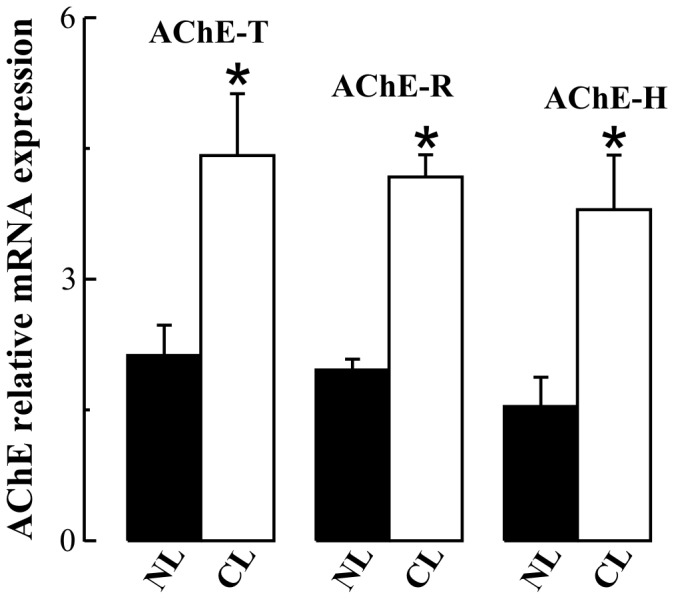
Increased levels of acetycholinesterase (AChE) mRNA variants in cirrhotic liver. Relative mRNA levels of the liver transcripts for AChE (T, R and H; labelled AChE-T, AChE-R and AChE-H in the figure) were analyzed by QRT-PCR in non-diseased (NL; ▪; n = 5) and cirrhotic liver cases (CL; □; n = 9). The selected primers were: AChE-T transcript: Fw: 5′-CTTCCTCCCCAAATTGCTC3-′, Rev: 5′-TCCTGCTTGCTGTAGTGGTC-3′; AChE-R transcript: Fw: 5′-CTTCCTCCCCAAATTGCTC-3′, Rev: 5′-GGGGAGAAGAGAGGGGTTAC-3′; AChE-H transcript: Fw: 5′-CAATGAGCCCCGAGACC-3′, Rev: 5′-GAGCCTCCGAGGCGGTG-3′; GAPDH: Fw: 5′-AGCCACATCGCTCAGACAC-3′, Rev: 5′-GCCCAATACGACCAAATCC-3′. Values were calculated using relative standard curves and normalized to GAPDH. Specificity of the PCR products was confirmed by dissociation curve analysis. *p<0.05.

Quantitative RT-PCR analysis of AChE mRNA in liver extracts from cirrhotic patients demonstrated an increase in levels of the T-transcript (which encodes subunits which produce monomeric and tetrameric forms), compared to controls (108% increase; *p* = 0.03; Fig. 3). Similarly, and in agreement with Western blot analysis data, levels of the R-transcript, which encodes monomeric soluble subunits, were also increased in cirrhotic livers compared to controls (113% increase; *p* = 0.01; Fig. 3). RT-PCR analysis was used to determine the mRNA levels for the minor splice variant, the AChE H-transcript. Levels of the H-transcript were also increased in the cirrhotic liver (146% increase; *p* = 0.03; Fig. 3). In summary, while enzyme levels remain unchanged, expression levels of the different AChE variants are substantially altered in liver cirrhosis.

**Figure 4:**
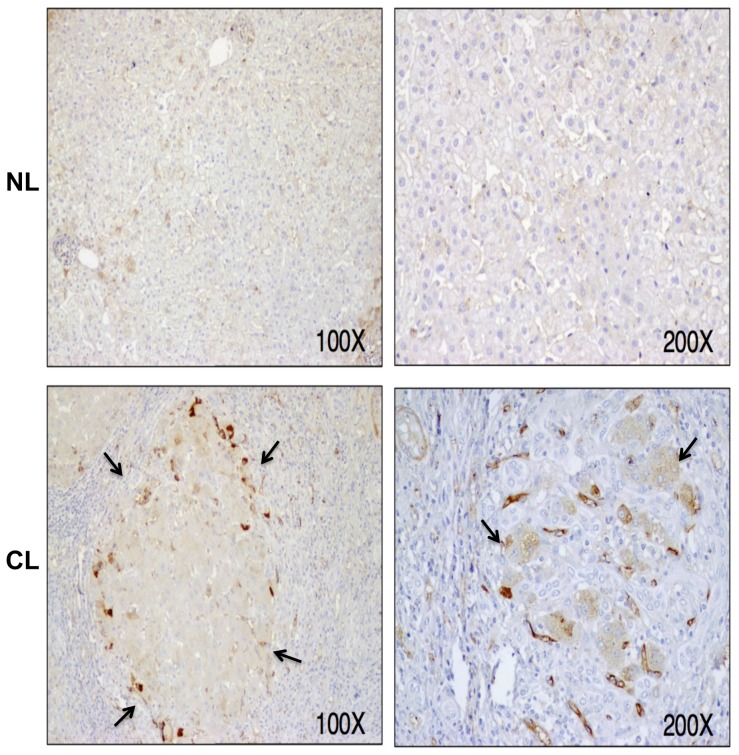
Immunopositive acetylcholinesterase (AChE) staining in cirrhotic human liver. Representative photographs of liver specimens from a control subject (NL) and a patient with hepatitis C virus (HCV)-induced cirrhosis (CL), stained for AChE (antibody N19). Weak immunopositive AChE was mainly localized in normal hepatocytes. This immunoreactivity was clearly increased in the cirrhotic liver, especially in hepatocytes preferentially located at borders surrounding regenerative liver nodules (arrows).

Finally, the distribution of AChE-positive cells in the rat liver is demonstrated by immunocytochemistry using the N-19 antibody. While AChE was barely detected in normal human livers, it was clearly observed in parenchymal cells in cirrhotic livers (Fig. 4), in particular, in hepatocytes at the periphery of regenerative nodules. Hepatic protein expression of the AChE subtypes could not be determined as no subtype-specific antibodies are available for immunocytochemistry.

## Discussion

In this study, a significant change in AChE was observed at both protein and mRNA levels in the cirrhotic human liver, while enzyme activity was unchanged compared to controls. Increase in AChE protein levels was also confirmed by immunohistological examination. Similarly, in the serum of cirrhotic subjects, AChE activity remained unchanged, while protein levels of the soluble AChE-R was increased. The increase in the level of AChE protein was associated by decrease in BChE activity in both serum and cirrhotic liver.

Serum BChE activity levels have been widely used as a test of liver function. Only few studies including a low number of patients have assessed cholinesterase levels in human liver [8], [9], and to the best of our knowledge, no study has included cirrhotic patients. In addition, conventional protein tissue extraction techniques grossly underestimated BChE activity as substantial inhibition by the presence of detergents occurs [25]. Because our main goal was to describe how AChE is regulated in the cirrhotic liver, we used the detergent Triton X-100 for cholinesterase extraction, which allows a recovery of 80–90% of the total tissue AChE activity [19]. Further studies should better assess the degree of BChE activity in the liver, since we can not rule out some inhibition by Triton X-100 [25]. Nonetheless, to our knowledge, this is the first report indicating that serum BChE decreases in t withhe livers of patients with end-stage liver disease (ie, cirrhosis).

We have previously described a marked decrease in serum and liver AChE levels, with no changes in BChE levels, in a cirrhotic rat model [13]. This change was associated with a selective loss of the tetrameric form of AChE. Thus, in cirrhotic conditions, the expression of both cholinesterases is differentially affected between humans and rats. These differences are not apparent when all the molecular forms of both cholinesterases are considered collectively. In the liver and serum, tetrameric forms and light species have been identified for both, AChE and BChE. However, the level of contribution of each form is dependent on the species being studied. In humans the amount of BChE in plasma is much higher than AChE, with a large amount of BChE tetramers and small amounts of the light AChE species; while in rat serum tetrameric AChE is the major cholinesterase species. The physiological significance of cholinesterase activity in liver and serum has thus far not been elucidated, but we can hypothesize that evolutionarily, the same role has been performed by tetrameric AChE in rats and by tetrameric BChE in humans. In both animal species the depletion of tetramers, G_4_ BChE in humans or G_4_ AChE in rats, is linked to chronic liver disease and probably reflects tissue damage, although the physiological relevance is still to be determined.

Despite the unchanged AChE activity in human cirrhotic liver and serum we observed an increase in the immunoreactivity to different anti-AChE antibodies by western blotting. This apparent increase in AChE protein has been corroborated by immunohistochemistry and, at expression level, is accompanied by an increase of AChE transcripts. The majority of increased AChE immunoreactivity is located within hepatocytes located at borders surrounding regenerative nodules. Interestingly, AChE has been proposed as a key predictor for hepatocellular carcinoma prognosis [12]. The possibility that AChE participates in the development of cancerous nodules requires further research. Beside a role in the process of normal and pathological cell proliferation, it is has been suggested that AChE might be also involve in the promotion of various types of apoptosis, [26], [27]. Moreover, a 55 kDa AChE protein results selectively induced during apoptosis [28]. Thus, it is plausible that AChE participates in hepatocellular apoptosis that characterizes cirrhosis. Further functional studies should confirm this hypothesis.

Two aspects that should also be considered are the non-catalytic nature of the increased AChE protein and the identity of the species. First, as both active and inactive subunits of the protein could contribute to the immunoreactivity of the bands, we can logically assume that enzymatically inactive AChE protein species are increased in the cirrhotic liver. AChE is present as both active and inactive subunits and the inactive AChE molecules have been described as lighter AChE molecules [14], [15], [16]. Interestingly, in the rat model of cirrhosis, despite the overall decrease in AChE activity due to tetramer depletion, we have also observed an increase in some immunoreactive AChE band, which can be attributed in part to inactive AChE, as well as to an increase of the R transcript [13], demonstrating again, similarities between the cirrhotic rat model and the disease in humans. The presence of an inactive catalytic pool of AChE, which is increased in the cirrhotic liver, suggests AChE may have roles in the liver independent of its enzymatic activity. Multiple activities of AChE include non-classical actions that are independent of the catalytic capacity, thus catalytically inactive AChE species may still be physiologically active. The inactive AChE fraction is abundant in embryonic tissues [2] where a cholinergic function has thus far not been attributed. It has also been demonstrated that transgenic overexpression of enzymatically inactive AChE can influence neurodevelopment [29]. Indeed, AChE levels are routinely estimated enzymatically and not immunochemically. As such, little information is available on this non-catalytic pool of AChE in both pathological and non-pathological conditions. Whether non-catalytic AChE in liver has physiological significance, and how it is affected during pathology, are issues that warrant further study.

It is also important to further define the nature of the AChE species which is increased in liver cirrhosis. AChE exhibits high molecular polymorphism contributed as a result of alternative splicing. AChE exists in different isoforms, with the same catalytic domain but with distinct C-terminal peptides. These splice variants may have different and specific functions [30], dependent on their specific subcellular localization. Alternatively, specific properties associated to the particular C-terminus of AChE may modulate binding affinities for other interacting proteins, etc. Our data demonstrates that the major liver AChE species, the 55 kDa subunits, correspond to both R- and T-subunits, as react with specific antibodies. Although the predicted molecular weight of the AChE subunits is ∼70 kDa in size, our as well as other studies strongly suggest the specificity identity of lower AChE molecular weight bands [10], [13], [21], [28], [31], [32]. The specificity of these AChE bands was confirmed by immunoblotting plasma samples with the anti-human AChE N-terminal antibody, N-19, and the T548 antibody. R- and T-subunits of this size are present in different human tissues with the exception of red-blood cells, which possess only H-subunits [10]. The presence of the H-subunit was not assessed in plasma due to the lack of specific antibodies. Analysis of the levels of liver AChE transcripts indicated that all AChE molecular isoforms appear to be similarly altered in liver cirrhosis. We were able to determine an increase in the AChE-R specie in cirrhotic serum, while levels of soluble AChE-T remain unaltered. The relatively rare AChE-R is an ubiquitous protein present at low levels in many, if not all, tissues (similar to the more common AChE-T specie); and is classically induced under psychological, chemical or physical stress [33]. The AChE-R can display distinct and sometimes an inverse function to the major AChE-T species (see for example [34]), and can also interact with specific partners such as the scaffold protein RACK1 [34], the kinase PKCbeta II [35], or the glycolytic enzyme enolase [34]. Over-expression of AChE-R has been associated with germ cell apoptosis [36], hematopoietic proliferation [37], and in abnormal cells correlated with cell proliferation [38]. In particular, AChE-R is elevated in various tumor types [39], and in other diverse pathological conditions such as oxidative stress [40] and behavioral impairment [41], among others. The significance of the increase in the stress-induced R-AChE in cirrhotic serum is unclear and further studies of the relationship between the AChE-R specie and liver pathology are still needed.

In conclusion, this is the first study to reliably demonstrate that liver disease affects the expression and levels of AChE in human liver and serum. The possibility to monitor changes not only in serum BChE activity, but also in the soluble AChE-R, as a function of liver disease progression and prognosis requires further consideration.

## Supporting Information

Figure S1 **Decreased levels of plasma butyrylcholinesterase (BChE) activity in cirrhotic individuals.** (**A**) Plasma from non-diseased controls (NL) and individuals with liver cirrhosis (CL) (same cases that in Fig. 1) assayed for BChE activity (before immunoprecipitation), displayed diminished levels in CL. (**B**) Sedimentation analysis displays a decrease in the major BChE tetrameric forms (G_4_) which decrease in CL (○), in comparison with normal liver (•).* *p*<0.05 significantly different from NL, as assessed by Student's t-test.
(TIF)

Figure S2 **Immunodetection of AChE subunits with the antibody T548.** (**A**) Representative immunoblot of samples from human plasma and cerebrospinal fluid (CSF), and from brain (frontal cortex), red blood cells (RBCs), and liver extracts from non-disease subjects were blotted with the T548 antibody. A representative immunoblot with the antibody N-19 from a human plasma sample is included (left panel). The antibody T548 confirms the specificity of the 66 and 55 kDa bands, whereas the 78 kDa subunit, revealed with the antibody N19, was non immunoreactive to the antibody T548. (**B**) The specificity of T458 has been demonstrated in brain extracts from wild-type and AChE knockout mice (see ref. [24]), and confirmed here by blotting cortical brain extracts from 11-month old AChE knockout mice (AChE^−/−^; see [42]) and age-matched wild type controls (AChE^+/+^) (equivalent amounts of protein were loaded in each lane).
(TIF)

Figure S3 **Decreased liver butyrylcholinesterase (BChE) activity in cirrhotic individuals.** (**A**) Levels of liver BChE from non-diseased subjects (NL) and cases with cirrhosis (CL) (same cases as in Fig. 2) measured prior to BChE depletion, displayed diminished levels in CL. * *p*<0.05. (**B**) The representative profiles of BChE molecular forms displays light monomers and dimers (G_1_+G_2_), but also tetramers (G_4_). The decrease in BChE activity in CL (○), in comparison with normal liver (•), corresponds to a decrease in all molecular forms.
(TIF)
