# Supplementary figures and images for: Readthrough Acetylcholinesterase Is Increased in Human Liver Cirrhosis
Source: PLoS One. 2012 Sep 13;7(9):e44598. doi: 10.1371/journal.pone.0044598 (PMC3441564; doi:10.1371/journal.pone.0044598)

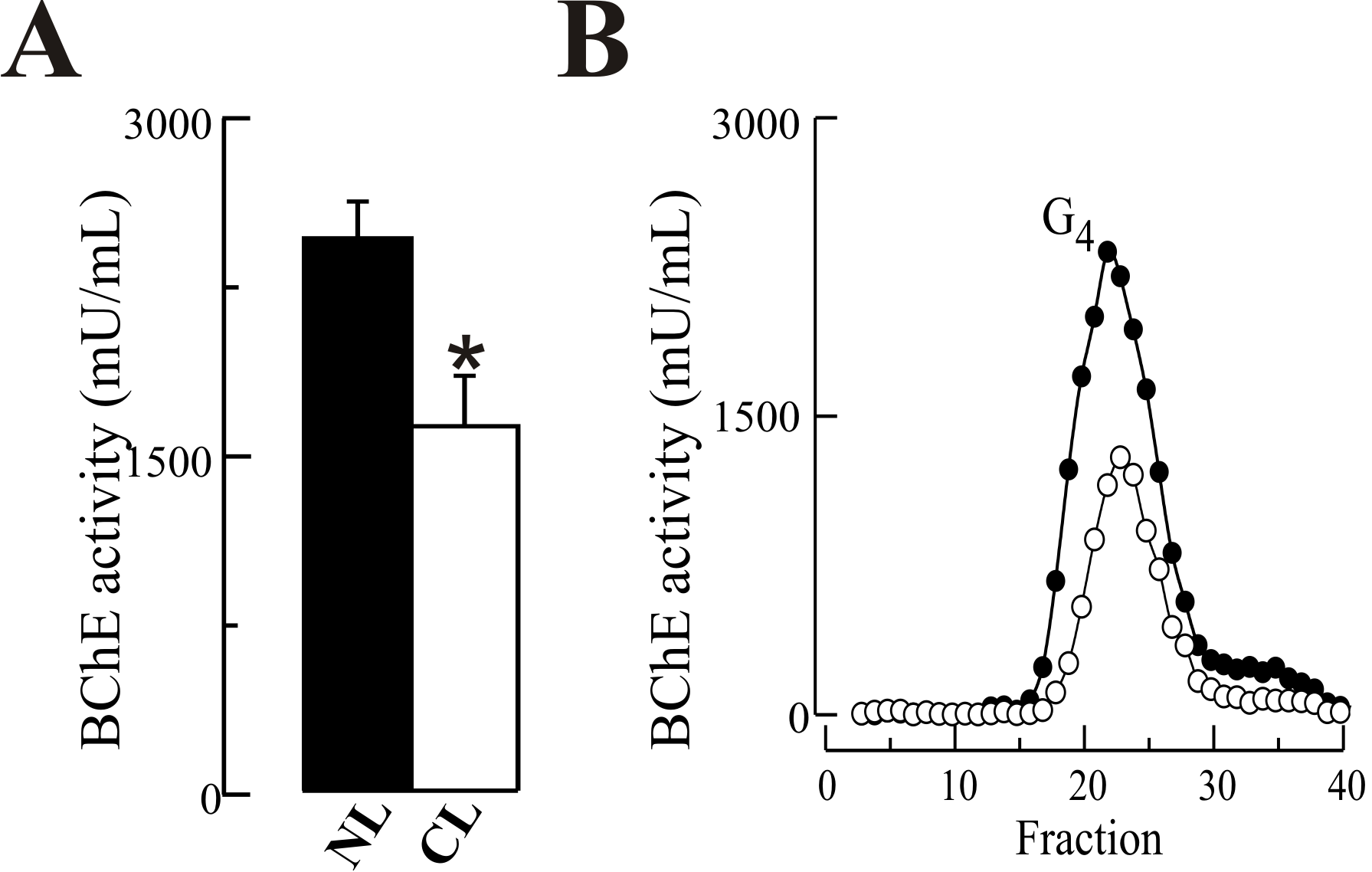

Supplement: Figure S1 — Decreased levels of plasma butyrylcholinesterase (BChE) activity in cirrhotic individuals. (A) Plasma from non-diseased controls (NL) and individuals with liver cirrhosis (CL) (same cases that in Fig. 1) assayed for BChE activity (before immunoprecipitation), displayed diminished levels in CL. (B) Sedimentation analysis displays a decrease in the major BChE tetrameric forms (G4) which decrease in CL (○), in comparison with normal liver (•).* p<0.05 significantly different from NL, as assessed by Student's t-test. (TIF) [file pone.0044598.s001.tif]

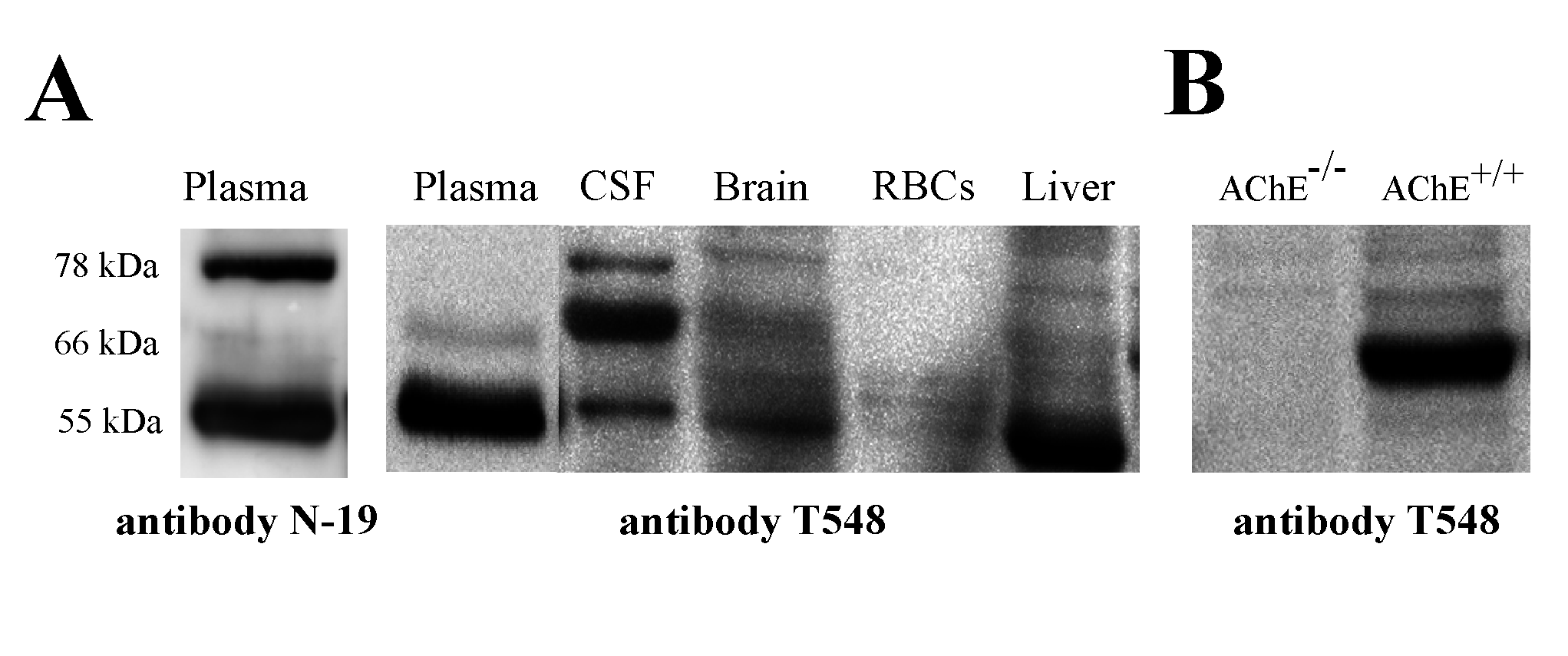

Supplement: Figure S2 — Immunodetection of AChE subunits with the antibody T548. (A) Representative immunoblot of samples from human plasma and cerebrospinal fluid (CSF), and from brain (frontal cortex), red blood cells (RBCs), and liver extracts from non-disease subjects were blotted with the T548 antibody. A representative immunoblot with the antibody N-19 from a human plasma sample is included (left panel). The antibody T548 confirms the specificity of the 66 and 55 kDa bands, whereas the 78 kDa subunit, revealed with the antibody N19, was non immunoreactive to the antibody T548. (B) The specificity of T458 has been demonstrated in brain extracts from wild-type and AChE knockout mice (see ref. [24]), and confirmed here by blotting cortical brain extracts from 11-month old AChE knockout mice (AChE−/−; see [42]) and age-matched wild type controls (AChE+/+) (equivalent amounts of protein were loaded in each lane). (TIF) [file pone.0044598.s002.tif]

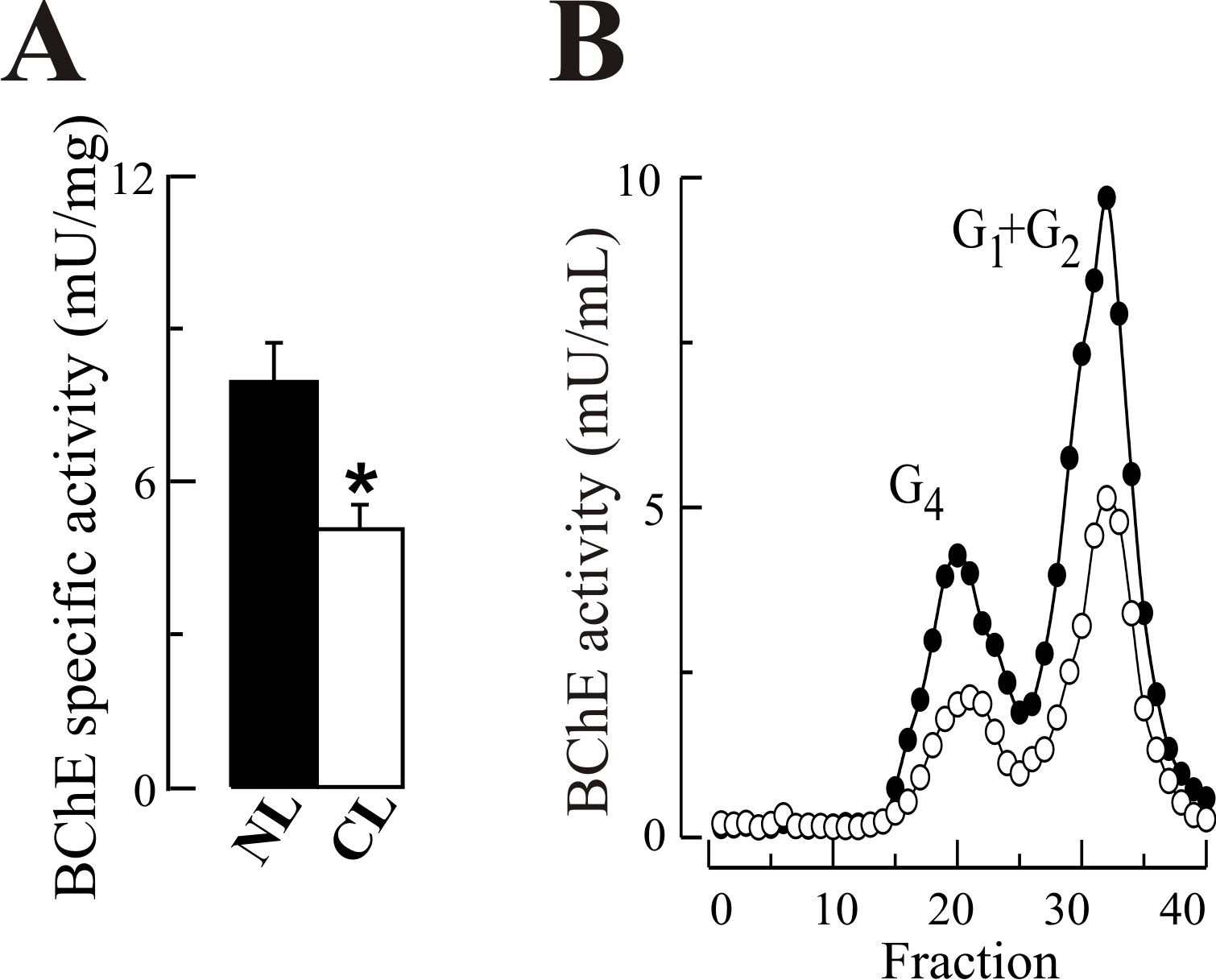

Supplement: Figure S3 — Decreased liver butyrylcholinesterase (BChE) activity in cirrhotic individuals. (A) Levels of liver BChE from non-diseased subjects (NL) and cases with cirrhosis (CL) (same cases as in Fig. 2) measured prior to BChE depletion, displayed diminished levels in CL. * p<0.05. (B) The representative profiles of BChE molecular forms displays light monomers and dimers (G1+G2), but also tetramers (G4). The decrease in BChE activity in CL (○), in comparison with normal liver (•), corresponds to a decrease in all molecular forms. (TIF) [file pone.0044598.s003.tif]
